# Direct Anastomosis Versus Conduit Repair for Right Ventricular Outflow Tract Reconstruction in Common Arterial Trunk: A Meta-Analysis of Reconstructed Time-to-Event Data

**DOI:** 10.1093/icvts/ivag029

**Published:** 2026-01-27

**Authors:** Abdullah Almehandi, Lucas Diniz, Enzzo Barrozo Marrazzo, Adriana Loricchio Veiga, Latefah Alotaibi, Yahya Ali, Abdulrahman O Al-Naseem, Hamood Al Kindi, Gunter Kerst, Tulio Caldonazo

**Affiliations:** Department of Cardiovascular Science, University College London, WC1E 6BT London, United Kingdom; Department of Medical Sciences, Federal Fluminense University, 24220-900 Rio de Janeiro, Brazil; Pontifical Catholic University of Minas Gerais, 37714-620 Poços de Caldas, Brazil; Universidad de Buenos Aires, C1053ABH Buenos Aires, Argentina; Department of Surgery, Jaber Al-Ahmad Hospital, 91710 Kuwait City, Kuwait; Faculty of Life Sciences and Medicine, King’s College London, WC2R 2LS London, WC2R 2LS, United Kingdom; Department of Orthopaedic Surgery, McGill University, H3A 0G4, Montréal, Canada; Department of Cardiothoracic Surgery, Sultan Qaboos University Hospital, 123 Sib, Oman; Clinic for Pediatric Cardiology and Congenital Heart Disease, Klinikum Stuttgart, 70174 Stuttgart, Germany; Department of Cardiothoracic Surgery, Jena University Hospital, Friedrich Schiller University of Jena, 07747 Jena, Germany

**Keywords:** common arterial trunk (CAT), direct anastomosis (DA), right ventricular outflow tract (RVOT)

## Abstract

**Objectives:**

Repair of common arterial trunk (CAT) involves establishing the right ventricular outflow tract (RVOT) using either a conduit or a direct right ventricle–pulmonary artery (RVPA) anastomosis (DA). Conduits offer a valved pathway but are limited by durability and availability. The comparative outcomes of these 2 techniques remain uncertain. This work assessed whether DA improves survival, reduces complications and reintervention outcomes compared to conduit repair.

**Methods:**

PubMed, Web of Science, EMBASE, and Cochrane Central were searched for studies comparing conduit versus DA for RVOT reconstruction from February 20, 2025 to March 30 30, 2025. The primary outcome was early mortality; secondary outcomes included haemodynamics, recovery, and complications. Time-to-event data were reconstructed from Kaplan-Meier curves. Pooled hazard ratios (HRs), risk ratios (RRs), or mean differences (MDs) with 95% confidence intervals (CIs) were calculated using random-effects models.

**Results:**

Eleven studies (767 patients; 419 conduit, 348 DA) were included. Early mortality (RR = 0.61, 95% CI, 0.26-1.44, *P* = .220) and long-term survival (HR = 1.11, 95% CI, 0.61-2.02, *P* = .738) were similar. Reoperation was more frequent in the conduit group (HR = 1.77, 95% CI, 1.05-3.01, *P* = .034). Conduit repair required longer ventilation (MD = 3.44 days, *P* = .010) and hospitalization (MD = 4.77 days, *P* = .030), with comparable ICU stay and RVOT growth. Truncal valve insufficiency (RR = 0.13, *P* = .130 for truncal valve vs conduit) was similar in incidence following DA.

**Conclusions:**

Conduit and DA repairs yield similar survival and postoperative complications in CAT, while DA offers fewer reoperations and faster recovery. Data from future prospective multicentre trials will support decision-making.

## Graphical abstract

**Figure:**
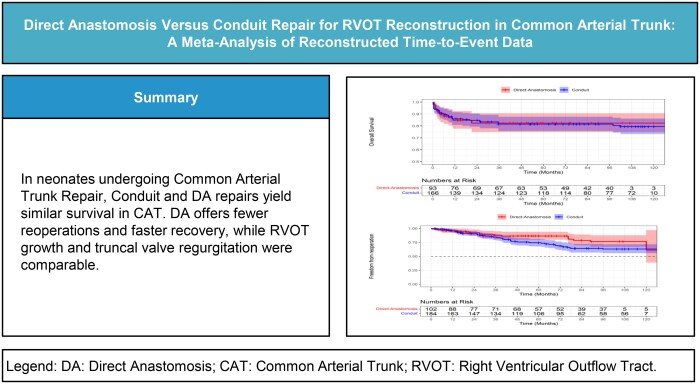

## INTRODUCTION

Common arterial trunk (CAT), also known as truncus arteriosus, is a rare congenital cardiac anomaly, accounting for approximately 0.21%-0.34% of all congenital heart defects.^1^ It is characterized by a single arterial trunk that arises from the ventricular mass and gives rise to the systemic, pulmonary, and coronary circulations. This anomaly also involves the right ventricular outflow tract (RVOT) and the great arteries.^2^ Without surgical intervention, CAT is uniformly fatal in early infancy due to the rapid development of congestive heart failure and pulmonary hypertension.^3^ Therefore, establishing a timely and well-structured surgical strategy is critical to improving survival outcomes.^4^^,^^5^

Two principal surgical techniques are used for right ventricular outflow tract (RVOT) reconstruction in CAT: conduit-based repair and direct right ventricle-to-pulmonary artery (RVPA) anastomosis. Although conduit repair has been the standard approach, its application is limited in neonates with small ventricles by the unavailability of appropriately sized conduits and complications related to xenografts, including early obstruction and suboptimal haemodynamics.^1^^,^^6^^,^^7^ In contrast, direct RVPA anastomosis resolves conduit availability issues and decreases early obstruction and reintervention incidence, but the absence of a functional pulmonary valve may adversely affect long-term pulmonary haemodynamics.^8^^,^^9^ The optimal surgical technique is therefore highly dependent on patient-specific anatomical and haemodynamic considerations.^1^

In light of these considerations, we undertook a meta-analysis and systematic review with quantitatively synthesized comparative evidence using reconstructed Kaplan-Meier data, to compare the outcomes of direct RVPA anastomosis to conduit-based repair, focusing on survival, complication rates, and the need for reintervention.

## METHODS

This systematic review and meta-analysis were conducted in accordance with the Preferred Reporting Items for Systematic Reviews and Meta-Analyses (PRISMA) guidelines.^10^ This review was registered with the National Institute for Health Research International Registry of Systematic Reviews (PROSPERO, CRD420251246981).

### Eligibility criteria

Inclusion in this meta-analysis was restricted to studies that met all the following eligibility criteria: (1) randomized trials or non-randomized cohorts; (2) comparing conduit to non-conduit RVPA direct anastomosis; (3) enrolling patients who underwent primary repair for common arterial trunk defect. In addition, studies were included only if, in the English language, they reported any of the clinical outcomes of interest. We excluded studies with (1) no control group; (2) reported outcomes not being stratified by conduit and non-conduit RVPA direct anastomosis, and (3) common arterial trunk patients undergoing staged repair, to avoid a mixed cohort.

### Search strategy and data extraction

We systematically searched for English-language articles in PubMed, Web of Science, Cochrane Central Register of Controlled Trials and EMBASE from February 20, 2025 to March 30, 2025 with a unified search strategy across all databases: “truncus arteriosus,” “common arterial trunk,” “persistent truncus,” “congenital heart disease,” “conotruncal anomaly,” “single arterial trunk,” “cyanotic CHD”; “conduit, homograft,” “valved conduit,” “tube/vascular/prosthetic graft,” “RV–PA conduit,” “outflow tract reconstruction”; “non-conduit,” “conduit-free,” “direct RV–PA connection,” “native anastomosis” (**Table S1**). Screening of the aforementioned medical databases, including references from included studies, previous systematic reviews, and meta-analyses were also conducted by 2 reviewers independently and blinded to each other’s decisions. Data extraction and quality assessment were likewise conducted independently with reviewer blinding.

### Quality assessment

Risk of bias was assessed for non-randomized studies via the ROBINS-I^11^; these studies were assessed against 7 domains, including: confounding, classification of interventions, selection of participants, deviation from the intended intervention, missing data, outcome measurement, and selection of the reported result. Risk assessment included low (green), moderate (yellow), and serious risk (red). Finally, publication bias was investigated by funnel-plot analysis of the primary outcome.

### Outcomes

Primary outcomes included: Kaplan-Meier analysis of overall survival, reoperation, and a pooled forest plot analysis for short-term mortality. Secondary data included: postoperative surgical reoperation, cardiopulmonary bypass (CPB) time, and cross-clamp time. Postoperatively, RVOT growth (mm), incidence of truncal valve regurgitation, hospital stay duration, ICU stay duration, and duration of mechanical ventilation.

### Kaplan-Meier curve analysis

A 2-column dataset containing time points and survival probabilities was extracted from each Kaplan-Meier curve using the “ScanIt” software, together with the associated number-at-risk tables. Subsequently, using R software version 4.4.3, the “IPDfromKM” package was employed to convert these coordinates into individual patient data (IPD) by applying the iterative algorithm proposed by Guyot et al.^12^ Subsequently, an object containing the individual patient data from all Kaplan-Meier curves, extracted from each article, was then used to generate a pooled Kaplan-Meier curve using the “survival” and “survminer” packages, specifically leveraging the “survfit” and “ggsurvplot” functions.

The accuracy of the reconstructed curves was validated using the root mean square error (RMSE) and the Kolmogorov-Smirnov test to compare the distribution of the reconstructed and original Kaplan-Meier curves. Additionally, a 2-sided log-rank test was performed to compare the “Direct-anastomosis” and “Conduit” arms for assessment of survival differences. Restricted mean survival time (RMST) was estimated at 12, 24, 48, and 96 months for both arms, providing an alternative summary measure in cases where the proportional hazards assumption was violated.

For the studies “Kai” and “Lacour,” censoring was assumed to be uniform due to the unavailability of number-at-risk tables. Reoperation outcomes were subject to informative censoring, including death and loss to follow-up, which may influence the interpretation of results.

In parallel, linear regression analyses were conducted for mortality and reoperation rates, comparing them with publication year and age (in days). The regression models were weighted by population size, included 95% confidence intervals (CIs) for the fitted curves, and reported *R*^2^ values, where values close to 1 indicate a strong association and values close to 0 indicate no association.

### Statistical analysis

Risk ratio (RR), mean difference (MD), and 95% CIs were calculated for each outcome. An OR greater than 1, or an MD greater than zero, corresponds to a greater frequency of the studied outcome. Continuous variables were analysed using MD and 95% CI. Inherent clinical heterogeneity between the studies was balanced via the implementation of a random effects model (Der Simonian-Laird). Results were displayed in forest plots.

Kaplan-Meier curve analysis was performed via the R program. Alpha error was set to 0.05. Between-study statistical heterogeneity was assessed with the Cochran Q statistic and by estimating *I*^2^. High heterogeneity was confirmed with a significance level of *P* < .10 and *I*^2^ of at least 50% or more. All statistical analyses were performed using Review Manager 5.3. A meta regression analysis was performed for early mortality and reoperation against the era of surgical reoperation and patient age, using fixed-effects weighted linear models.

## RESULTS

### Study characteristics

**Figure 1.**
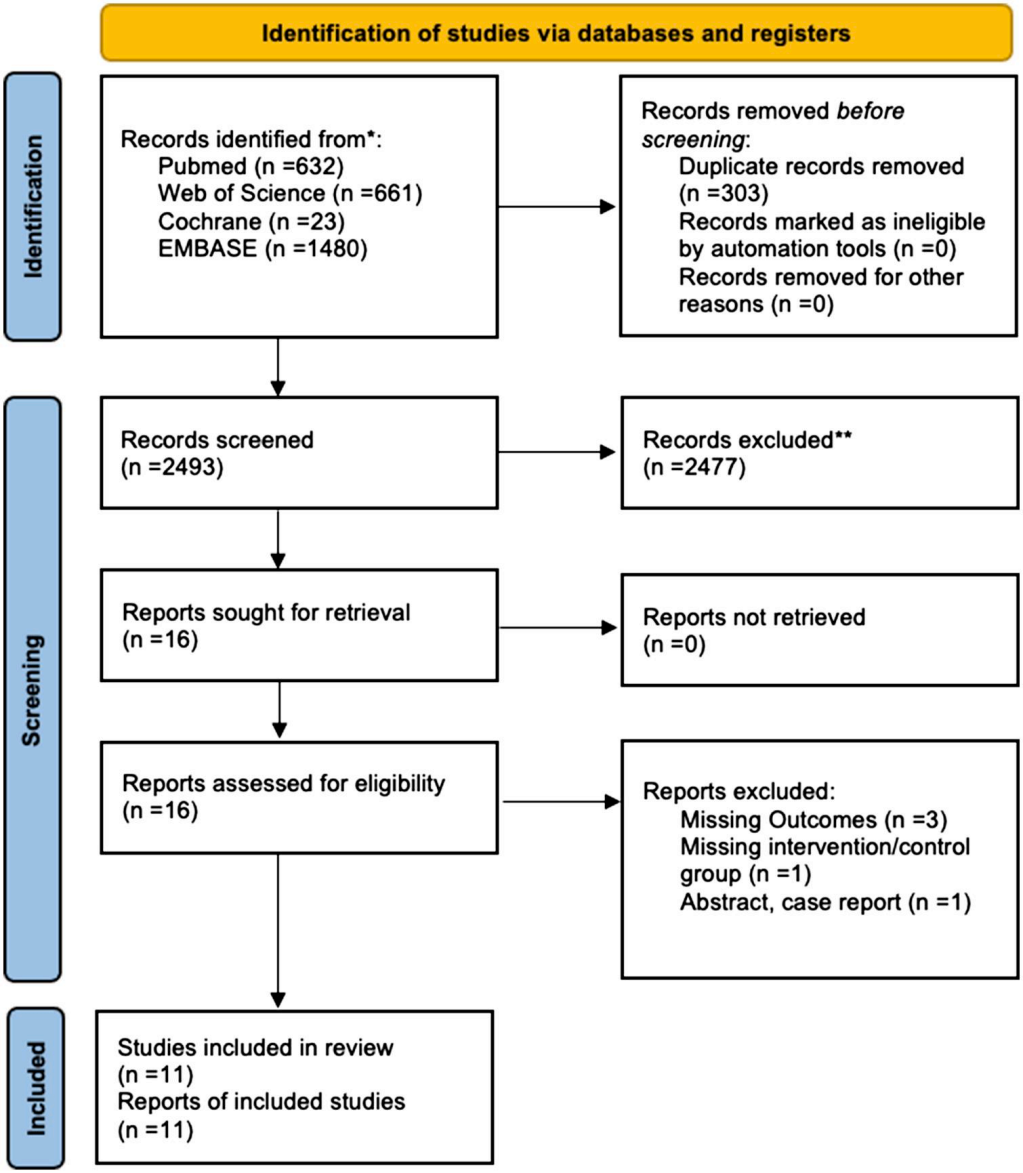
PRISMA Flow Diagram for Study Screening

A total of 2493 studies were retrieved from the systematic search, of which 11 met the criteria for inclusion in the final analysis.^9^^,^^13–21^  **Figure 1** shows the PRISMA flowchart for study selection. Included studies were published between 2009 and 2025; all 11 studies included were observational trials.

**Table 1** shows the details of the included studies. A total of 767 patients, 419 conduit, 348 DA patients were included in the final analysis. The number of patients in each study ranged from 12 to 75.

**Table 1.** Summary of Included Studies

Author	Year	Country	Study design	Follow-up (months)	Reported outcomes
Lacour-Gayet	1996	France	Retrospective observational single-centre study	38.0	Hospital mortality, survival, reoperations, pulmonary regurgitation, CPB time, and RVOT techniques.
Brown	2001	USA	Retrospective observational single-centre study	59.1	Hospital mortality, early survival, late death, reoperation for RVOT obstruction, type of RVOT reconstruction, functional status, medication use, and freedom from reoperation.
Danton	2001	UK	Retrospective observational single-centre study	50.0	Hospital and late mortality, surgical and percutaneous reintervention, risk factors, survival, freedom from reoperation, complications.
Chen	2005	US	Retrospective observational single-centre study	55.2	Mortality, reoperations, catheter interventions (CBI), outflow tract obstruction, pulmonary valve insufficiency, and ventricular function.
Raisky	2009	France	Retrospective observational single-centre study	40.0	Hospital mortality, late survival, freedom from reoperation; RV–PA gradient, RV/LV pressure ratio, CT-scan indices (Nakata, ostial index, RVOT growth).
Xu	2010	China	Retrospective observational single-centre study	49.7	Hospital mortality, actuarial survival, need for RVOT revision, growth and development of the right ventricular outflow tract, pulmonary artery growth, cardiac function, blood flow velocity at anastomosis/orifices, truncal valve morphology, severity of truncal valve regurgitation, and need for truncal valve reintervention.
Luo	2018	China	Retrospective observational single-centre study	55.8	Early and late mortality, intubation time, ICU, reoperations, RVOT growth, RVPA gradient, NYHA functional class
Derridji	2020	France	Retrospective observational single-centre study	127.2	Survival, mortality, reintervention, freedom from reintervention, and risk factors for mortality and reintervention.
Padalino	2020	Italy	Retrospective observational single-centre study	139.2	Survival, reoperations, reinterventions, adverse events (AEs), RV dysfunction/dilation, and length of hospital stay.
Moodley	2024	South Africa	Retrospective observational single-centre study	96.0	In-hospital mortality, reoperations, infectious and cardiac complications, and CPB time.
Reddy	2024	India	Retrospective observational single-centre study	97.8	Early and late mortality, reintervention, right ventricular dysfunction, conduit obstruction, and valve regurgitation.

Abbreviation: AE = adverse event; APBF = antegrade pulmonary blood flow; BDG = bidirectional Glenn; CBI = catheter-based intervention; CPB = cardiopulmonary bypass; ICU = intensive care unit; LV = left ventricle; MPAP = mean pulmonary artery pressure; NIRS = near-infrared spectroscopy; NYHA = New York Heart Association; RV = right ventricle; RVOT = right ventricular outflow tract; RVPA = right ventricle-to-pulmonary artery; SpO_2_ = systemic oxygen saturation; SVC = superior vena cava.

### Patient characteristics

**Table S2** summarizes the demographic data of the patient population in each study. The proportion of patients who underwent common arterial trunk repair with conduit placement varied between 20% and 90%. The mean age of patients ranged from 14.8 to 608.1 days old, and the percentage of males ranged from 22% to 76.9%. The mean weight ranged from 3.5 to 9.4 kg. Various truncus arteriosus classifications (I-IV) were observed in the patient population. The prevalence of interrupted aortic arch (IAA) ranged from 3.5% to 24%. Coronary artery anomalies were prevalent in 5.3%-47% of patients. Finally, weighted averages for pooled patients characteristics are summarized in **Table S3**.

### Quality assessment

In terms of ROBINS-I assessment (**Table S4**), a serious risk of bias was identified in Lacour-Gayet et al. (1996) and Moodley et al. (2024) mainly due to confounding risk and missing data. A moderate risk of bias was identified in Chen et al. (2005), Danton et al. (2001), Derridji et al. (2020), Moodley et al. (2024), Raisky et al. (2009), Reddy et al. (2024), and Xu (2010) for reasons that were mainly pertaining to risk of confounding, missing data, and bias in measurement and selection of the reported results.

Publication bias assessment of our primary outcome (short-term Mortality) is presented in **Figure S1**. Seven studies reported short-term mortality (Chen 2005, Lacour-Gayet 1995, Luo 2018, Danton 2001, Moodley 2024, Raisky 2009, and Reddy 2024). In this plot, the distribution is asymmetric. The left side, representing studies with a reduced risk, is sparsely populated, while smaller, less precise studies cluster on the right, indicating an increased risk. This pattern suggests publication bias, where studies showing no effect or an opposite effect may be missing. The outlier study, Reddy 2024, is a large, highly precise study with a strong positive effect. Its position strongly influences the pooled estimate. Overall, the asymmetry and missing studies indicate that the pooled effect may be overestimated and should be interpreted with caution.

### Pooled analysis

#### Primary outcomes: reconstructed data

**Figure 2.**
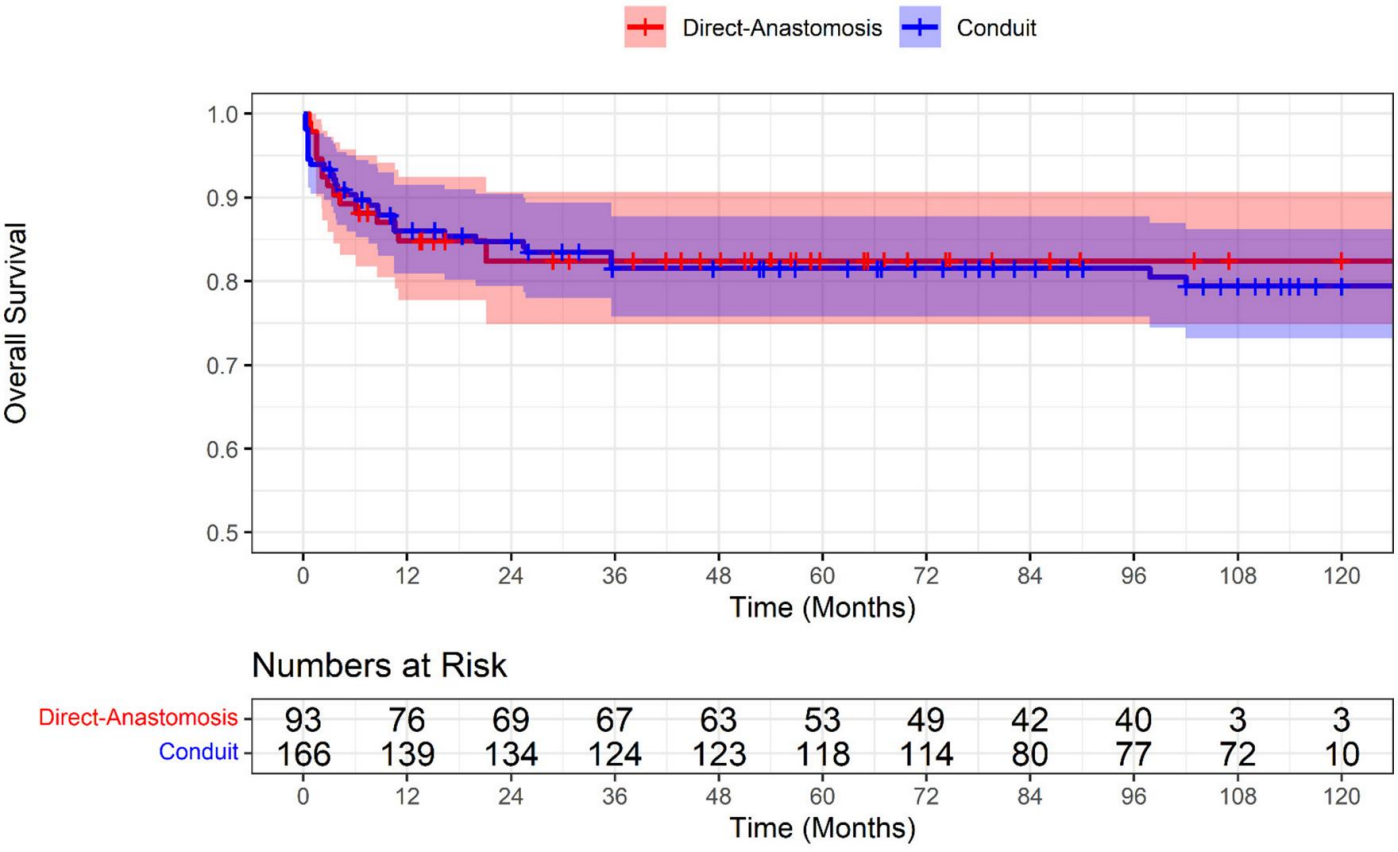
Kaplan-Meier Curve for Overall Survival Comparing Direct Anastomosis Versus Conduit Repair

The Kaplan-Meier curve in **Figure 2** shows no significant difference in overall survival over time between patients undergoing the Direct-Anastomosis and Conduit procedures (*P* = .738). The survival curves for the 2 groups are largely overlapping (hazard ratio, HR = 1.11 [95% CI, 0.61-2.02, *P* = .738]), in addition, the CI limits reflect statistical indifference.

**Figure 3.**
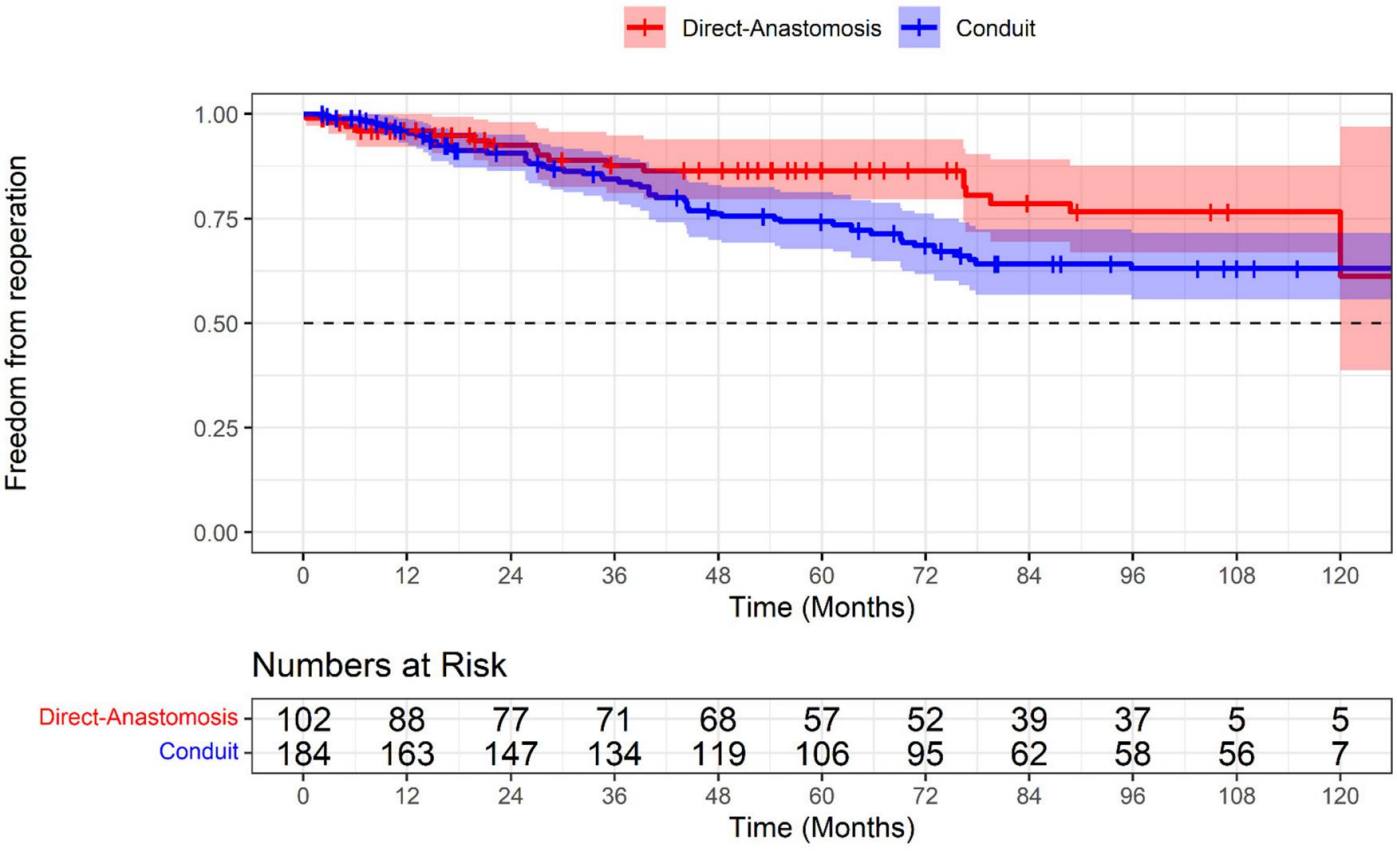
Kaplan-Meier Curve for Reoperation Post-Truncal Valve Repair Comparing Direct Anastomosis Versus Conduit Repair

The Kaplan-Meier curve in **Figure 3** shows a significant difference in the rate of reoperation over time between the 2 surgical groups. The curve for the Conduit group shows a higher probability of reoperation compared to the Direct-Anastomosis group. This visual interpretation is confirmed by the provided statistics, with patients in the Conduit group having a significantly increased risk of reoperation (HR = 1.77, 95% CI, 1.05-3.01, *P* = .034).

**Figure 4.**
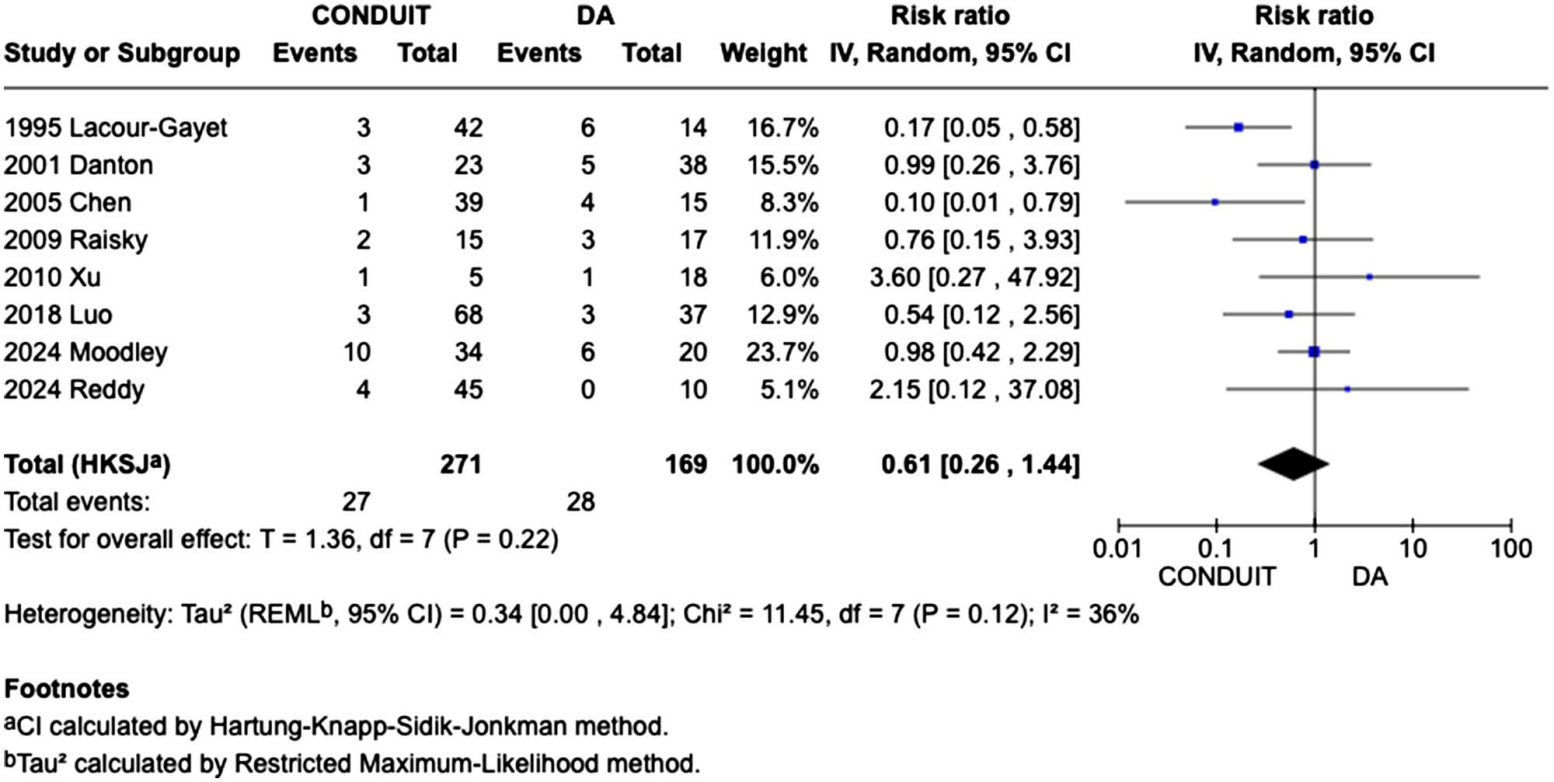
Forest Plot Presenting Pooled Data for Early Mortality

**Figure 4** shows the forest plot of early outcomes demonstrated comparable short-term mortality in the Non-conduit group compared to the Conduit group (RR = 0.61, 95% CI, 0.26-1.44, *P* = .220; *I*^2^ = 36%, *P *= .12) across 8 studies (*N* = 440; Conduit = 271, Non-conduit = 169).

#### Secondary outcomes

**Table 2** outlines the detailed secondary outcome results of the meta-analysis. When compared to the DA group, patients undergoing conduit repair had a comparable incidence of surgical reoperation postoperatively (OR= 1.84, *P =* .390) (**Figure S2**). In terms of operative parameters, patients undergoing conduit repair had a comparable CPB and cross-clamp times (min) to those undergoing direct anastomosis (MD= 15.82; *P* = .650) (**Figure S3**) and (MD = −8.34; *P *= .550) (**Figure S4**), respectively.

**Table 2.** A Summary of Pooled Outcomes Reported

Outcome	Number of studies	Number of patients	Effect estimate (95% CI, *P*-value)
Early mortality	8	440	RR = 0.61, 95% CI, 0.26–1.44, *P* = .22
Overall surgical reoperation	6	409	RR = 2.35; 95% CI, 0.39–14.18, *P* = .35
CPB time (mins)	4	252	MD = 15.82; 95% CI, –53.09 to 84.73, *P* = .65
Cross-clamp time (mins)	3	177	MD = −8,34; 95% CI, −35.42 to 18.73, *P* = .55
ICU LOS (days)	3	191	MD = 5.72; 95% CI, –1.79 to 13.23, *P* = .14
Hospital LOS (days)	**3**	**208**	**MD = 4.77; 95% CI, 0.48–9.05, *P* = .03**
Mechanical ventilation duration (days)	**3**	**191**	**MD = 3.44; 95% CI, 0.78–6.09, *P* = .01**
RVOT growth (mm)	3	160	MD = –2.81 mm; 95% CI, –7.84 to 2.22, *P* = .27
Truncal valve regurgitation	**4**	**241**	**RR = 0.25; 95% CI, 0.12–0.50, *P* < .0001**
Postoperative sepsis	3	169	RR = 1.68; 95% CI, 0.31 to 9.14, *P* = .55

Abbreviation: CI = confidence interval; CPB = cardiopulmonary bypass; ICU = intensive care unit; LOS = length of stay; MD = mean difference; OR = odds ratio; RR = risk ratio; RV = right ventricle; RVOT = right ventricular outflow tract; RV-PA = right ventricle-to-pulmonary artery. Bold outcomes showed statistically significant findings.

Recovery parameters, including ICU LOS, hospital LOS (days), and mechanical ventilation duration (min), showed varying results. While the duration of ICU recovery showed no significant difference between the groups undergoing conduit or direct anastomosis (MD = 5.72, *P *= .140) (**Figure S5**), the duration of hospital stay was significantly longer in the conduit group compared to direct repair (MD= 4.77, *P *= .030) (**Figure S6**). Finally, the duration of mechanical ventilation displayed significantly longer ventilation duration in the conduit group (MD = 3.44; *P *= .010) (**Figure S7**).

Importantly, postoperative RVOT growth (mm) was comparable between the DA and conduit repair group (MD: −2.81; *P *= .270) (**Figure S8**). The final remaining pooled postoperative complication postoperative incidence of truncal valve regurgitation was similar between the 2 cohorts (**Figure S9**).

#### Meta regression

Meta-regression analysis was insignificant for both year of study and patient’s age when analysed against short-term mortality (coefficient = 0.1438; *P* = .354) and (coefficient = 0.0267; *P* = .727) respectively (**Figures S10 and S11**, **Table S5**).

Furthermore, analysis for year of study and patient’s age against risk of future reoperation following primary repair was insignificant (coefficient = 0.4842; *P* = .192) and (coefficient = 0.0254; *P* = .798) respectively (**Figures S12 and S13**, **Table S6**).

## DISCUSSION

In this systematic review and meta-analysis of 11 studies including 767 (419 conduit) and 348 direct anastomosis patients, we compared outcomes in patients undergoing RVPA connection for common arterial trunk repair defects. The main findings from the pooled analyses were as follows: (1) early mortality and freedom from death across 120 months of follow-up were comparable, the latter achieved via pooled Kaplan-Meier analysis. (2) The non-conduit group reported greater freedom from reoperation at 5 years; Kaplan-Meier analysis indicated that this benefit in the non-conduit group became apparent after the first 24 months postoperatively. (3) Cardiopulmonary bypass (CPB) time and cross-clamp time were comparable between the 2 surgical strategies. (4) The conduit group experienced a greater association with longer duration of postoperative mechanical ventilation, a longer duration of hospital recovery, despite a comparable duration of ICU stay. (5) Postoperatively, RVOT growth (mm) and truncal valve regurgitation were comparable.

Current literature presents conflicting evidence on mortality, with some studies indicating equivalent survival for direct connection and others documenting increased risk.^9^^,^^14^^,^^15^^,^^17^^,^^18^^,^^22^ The similarity in mortality between groups in contemporary series may be attributed to improved perioperative management, including the use of nitric oxide and aggressive sedation strategies to prevent pulmonary hypertensive crises.^15^^,^^23^^,^^24^ Alternatively, earlier studies that found higher mortality with direct repair may reflect a less experienced era, as those procedures were often performed on older infants with higher pulmonary pressures and before modern management protocols were established.^18^^,^^22^ In contrast to earlier studies,^18^^,^^22^ our more recent pooled analysis found no significant difference for early in-hospital mortality. Furthermore, a pooled Kaplan-Meier analysis confirmed this, showing no statistically or visually meaningful difference in overall long-term survival (log-rank *P* = .74). Reported independent risk factors of mortality include coronary anomalies and CAT with interrupted aortic arch^1^^,^^17^^,^^25–30^

Kaplan-Meier analysis of reoperation showed a steeper decline in freedom from reoperation for the conduit group (∼65% at 10 years) compared to the non-conduit group (∼78% at 10 years; *P* = .036). While homograft and porcine valved conduits offer good survival, freedom from reoperation remains unsatisfactory, particularly in small infants with truncus arteriosus.^31–35^ This is a result of the direct anastomosis technique’s main advantage being the growth potential of its autologous posterior wall, which imaging studies confirm leads to significant RVOT and pulmonary artery growth, explaining its lower reoperation rate.^36^ Although reoperations can be performed with low mortality, the cumulative haemodynamic burden and repeat surgical trauma likely have negative long-term consequences for right ventricular function.^37^ Furthermore, an important potential confounding factor to point out is the characteristic anatomy, for example IAA or Coronary anomalies, which in some studies was more frequent in the conduit group (Padalino et al. and Raisky et al.), this added complexity to patient anatomy may bias results such as hospital length of stay and incidence of reoperation and explain their greater incidence in the conduit group.

Analysis of operative parameters showed that cardiopulmonary bypass, cross-clamp, were all comparable, indicating no inherent technical advantage for either approach. It has been reported in literature, however, that the direct anastomosis procedure is technically more demanding.^9^ In terms of haemodynamics postoperatively, an ideal repair should achieve optimal haemodynamics with a low gradient, a functional valve, and RVOT growth potential.^9^^,^^21^ Our pooled analysis reflects no haemodynamic advantage with a comparable RVOT growth and truncal valve regurgitation incidence postoperatively.

Our pooled analysis reporting comparable postoperative RVOT growth between the 2 techniques is, however, based on limited data from only 3 studies with a mean follow-up of 57 months. Our results contrast with other reports in the literature that reflect the superiority of the direct anastomosis technique for achieving greater RVOT growth.^18^^,^^38^ An RVOT reconstruction with growth potential provides a theoretically distinct advantage, as it could delay the need for reintervention.^36^

Pooled analysis of postoperative truncal valve regurgitation shows that the incidence of postoperative truncal valve regurgitation was comparable between the 2 groups. According to published literature, severe truncal valve regurgitation (TVR) is associated with early mortality, in part because its preoperative severity can be underestimated when assessed under systemic loading conditions (https://pmc.ncbi.nlm.nih.gov/articles/PMC5819734/). For treatment, multicentre experience indicates that valvuloplasty provides better early postoperative outcomes than valve replacement in children.^29^^,^^39^ However, in some patients with a preoperative dysplastic valve, regurgitation may worsen over time, ultimately necessitating aortic valve replacement.^40^

### Study limitations

The major limitations of this study include the retrospective, non-randomized, single-centre nature and small cohort size of included studies, attributable to the rarity of the malformation, a short study period, and high mortality rates. Furthermore, there was heterogeneity between the groups regarding surgical techniques, and the conduitless procedure was selectively used for less complex cases. In addition, assessment of publication bias was limited to the use of funnel plot generation via Review Manager, an assessment of small-study effects using DOI plots, the LFK indexes, or quantitative analysis via Egger’s regression test/Trim-and-fill test was not possible due to statistical software limitations. The comparative analysis was also challenged by significantly different follow-up times due to the recency of the conduitless technique and by incomplete follow-up data, which limits the generalizability of the findings.

## CONCLUSION

Conduit and DA repairs yield similar survival and postoperative complications in CAT, while DA offers fewer reoperations and faster recovery. Data from future prospective multicentre trials will support decision-making.

## Supplementary Material

ivag029_Supplementary_Data

## Data Availability

The data underlying this article are available in the article and in its online supplementary material.
