# Supplementary material for: Direct Anastomosis Versus Conduit Repair for Right Ventricular Outflow Tract Reconstruction in Common Arterial Trunk: A Meta-Analysis of Reconstructed Time-to-Event Data
Source: Interdiscip Cardiovasc Thorac Surg. 2026 Jan 27;41(2):ivag029. doi: 10.1093/icvts/ivag029 (PMC12927426; doi:10.1093/icvts/ivag029)

**Supplementary Material**

**Supplementary Table 1.** MEDLINE Search Strategy

**Supplementary Table 2.** The demographic data of the patient population in each study

**Supplementary Table 3.** Weighted Averages for baseline patient characteristics

**Supplementary Table 4.** ROBINS-1 Risk of Bias assessment

**Supplementary Table 5.** Meta regression of 30-day mortality.

**Supplementary Table 6.** Meta regression of Incidence of Surgical Reoperation.

**Supplementary Figure 1.** Publication Bias Assessment – Funnel Plot of the Primary Outcome.

**Supplementary Figure 2.** Forest plot for Postoperative Surgical Reoperation. DA= Direct Anastomosis, OR= odds ratio, CI= confidence interval.

**Supplementary Figure 3.** Forest plot for Cardiopulmonary Bypass (CPB) Time. DA= Direct Anastomosis, MD= mean difference, CI= confidence interval.
**Supplementary Figure 4.** Forest plot for Cross-Clamp Time. DA= Direct Anastomosis, MD= mean difference, CI= confidence interval.
**Supplementary Figure 5.** Forest plot for ICU Length of Stay. DA= Direct Anastomosis, MD= mean difference, CI= confidence interval.

**Supplementary Figure 6.** Forest plot for Hospital Length of Stay. DA= Direct Anastomosis, MD= mean difference, CI= confidence interval.
**Supplementary Figure 7.** Forest plot for Mechanical Ventilation Duration. DA= Direct Anastomosis, MD= mean difference, CI= confidence interval.
**Supplementary Figure 8.** Forest plot for RVOT Growth. DA= Direct Anastomosis, MD= mean difference, CI= confidence interval.
**Supplementary Figure 9.** Forest plot for Postoperative Truncal Valve Regurgitation. DA= Direct Anastomosis, OR= odds ratio, CI= confidence interval.
**Supplementary Figure 10.** Meta-regression plot for Mortality and Year.

**Supplementary Figure 11.** Meta-regression plot for Mortality and Age.

**Supplementary Figure 12.** Meta-regression plot for Incidence of Surgical Reoperation and Year.

**Supplementary Figure 13.** Meta-regression plot for Incidence of Surgical Reoperation and Age.

**Supplementary Table 1.** Search strategy used for Pubmed, Web of Science, EMBASE and Cochrane Databases.

| Search:  ("truncus arteriosus" OR "common arterial trunk" OR "persistent truncus" OR "truncus" OR "congenital heart disease" OR "congenital heart defect" OR "conotruncal anomaly" OR "single arterial trunk" OR "cyanotic congenital heart disease") AND ("conduit" OR "homograft" OR "graft" OR "valved conduit" OR "tube graft" OR "vascular graft" OR "synthetic conduit" OR "prosthetic graft" OR "RV to PA conduit" OR "RVPA conduit" OR "outflow tract reconstruction") AND ("non conduit" OR "non-conduit" OR "conduit free" OR "conduit-free" OR "direct RV to PA" OR "direct RVPA" OR "RVPA without conduit" OR "native connection" OR "native anastomosis" OR "direct anastomosis" OR "right ventricle pulmonary artery connection") |
| --- |
| **Translations** |
| ("truncus arteriosus"[MeSH Terms] OR ("truncus"[All Fields] AND "arteriosus"[All Fields]) OR "truncus arteriosus"[All Fields] OR ("truncus arteriosus, persistent"[MeSH Terms] OR ("truncus"[All Fields] AND "arteriosus"[All Fields] AND "persistent"[All Fields]) OR "persistent truncus arteriosus"[All Fields] OR ("common"[All Fields] AND "arterial"[All Fields] AND "trunk"[All Fields]) OR "common arterial trunk"[All Fields]) OR (("persist"[All Fields] OR "persistance"[All Fields] OR "persistant"[All Fields] OR "persisted"[All Fields] OR "persistence"[All Fields] OR "persistences"[All Fields] OR "persistencies"[All Fields] OR "persistency"[All Fields] OR "persistent"[All Fields] OR "persistently"[All Fields] OR "persistents"[All Fields] OR "persister"[All Fields] OR "persisters"[All Fields] OR "persisting"[All Fields] OR "persists"[All Fields]) AND ("torso"[MeSH Terms] OR "torso"[All Fields] OR "truncus"[All Fields])) OR ("torso"[MeSH Terms] OR "torso"[All Fields] OR "truncus"[All Fields]) OR ("heart defects, congenital"[MeSH Terms] OR ("heart"[All Fields] AND "defects"[All Fields] AND "congenital"[All Fields]) OR "congenital heart defects"[All Fields] OR ("congenital"[All Fields] AND "heart"[All Fields] AND "disease"[All Fields]) OR "congenital heart disease"[All Fields]) OR ("heart defects, congenital"[MeSH Terms] OR ("heart"[All Fields] AND "defects"[All Fields] AND "congenital"[All Fields]) OR "congenital heart defects"[All Fields] OR ("congenital"[All Fields] AND "heart"[All Fields] AND "defect"[All Fields]) OR "congenital heart defect"[All Fields]) OR ("conotruncal"[All Fields] AND ("abnormalities"[MeSH Subheading] OR "abnormalities"[All Fields] OR "anomalies"[All Fields] OR "anomalie"[All Fields] OR "anomaly"[All Fields])) OR (("single person"[MeSH Terms] OR ("single"[All Fields] AND "person"[All Fields]) OR "single person"[All Fields] OR "single"[All Fields] OR "singles"[All Fields]) AND ("arterialization"[All Fields] OR "arterializations"[All Fields] OR "arterialize"[All Fields] OR "arterialized"[All Fields] OR "arterializing"[All Fields] OR "arterially"[All Fields] OR "arterials"[All Fields] OR "arterie"[All Fields] OR "arteries"[MeSH Terms] OR "arteries"[All Fields] OR "arterial"[All Fields] OR "arteris"[All Fields] OR "artery"[All Fields] OR "arterious"[All Fields] OR "artery s"[All Fields] OR "arterys"[All Fields]) AND ("torso"[MeSH Terms] OR "torso"[All Fields] OR "trunk"[All Fields] OR "trunk s"[All Fields] OR "trunks"[All Fields])) OR (("cyanotic"[All Fields] OR "cyanotics"[All Fields]) AND ("heart defects, congenital"[MeSH Terms] OR ("heart"[All Fields] AND "defects"[All Fields] AND "congenital"[All Fields]) OR "congenital heart defects"[All Fields] OR ("congenital"[All Fields] AND "heart"[All Fields] AND "disease"[All Fields]) OR "congenital heart disease"[All Fields]))) AND ("conduit"[All Fields] OR "conduit s"[All Fields] OR "conduits"[All Fields] OR ("allografts"[MeSH Terms] OR "allografts"[All Fields] OR "homograft"[All Fields] OR "homografts"[All Fields]) OR ("graft s"[All Fields] OR "grafted"[All Fields] OR "graftings"[All Fields] OR "transplantation"[MeSH Subheading] OR "transplantation"[All Fields] OR "grafting"[All Fields] OR "transplantation"[MeSH Terms] OR "grafts"[All Fields] OR "transplants"[MeSH Terms] OR "transplants"[All Fields] OR "graft"[All Fields]) OR (("valve"[All Fields] OR "valve s"[All Fields] OR "valved"[All Fields] OR "valves"[All Fields] OR "valving"[All Fields]) AND ("conduit"[All Fields] OR "conduit s"[All Fields] OR "conduits"[All Fields])) OR ("tube"[All Fields] AND ("graft s"[All Fields] OR "grafted"[All Fields] OR "graftings"[All Fields] OR "transplantation"[MeSH Subheading] OR "transplantation"[All Fields] OR "grafting"[All Fields] OR "transplantation"[MeSH Terms] OR "grafts"[All Fields] OR "transplants"[MeSH Terms] OR "transplants"[All Fields] OR "graft"[All Fields])) OR ("vascular grafting"[MeSH Terms] OR ("vascular"[All Fields] AND "grafting"[All Fields]) OR "vascular grafting"[All Fields] OR ("vascular"[All Fields] AND "graft"[All Fields]) OR "vascular graft"[All Fields]) OR (("synthetic"[All Fields] OR "synthetically"[All Fields] OR "synthetics"[All Fields] OR "synthetize"[All Fields] OR "synthetized"[All Fields] OR "synthetizing"[All Fields]) AND ("conduit"[All Fields] OR "conduit s"[All Fields] OR "conduits"[All Fields])) OR (("prosthetic"[All Fields] OR "prosthetically"[All Fields] OR "prosthetics"[All Fields]) AND ("graft s"[All Fields] OR "grafted"[All Fields] OR "graftings"[All Fields] OR "transplantation"[MeSH Subheading] OR "transplantation"[All Fields] OR "grafting"[All Fields] OR "transplantation"[MeSH Terms] OR "grafts"[All Fields] OR "transplants"[MeSH Terms] OR "transplants"[All Fields] OR "graft"[All Fields])) OR ("RV"[All Fields] AND ("pathology"[MeSH Subheading] OR "pathology"[All Fields] OR "pa"[All Fields]) AND ("conduit"[All Fields] OR "conduit s"[All Fields] OR "conduits"[All Fields])) OR ("RVPA"[All Fields] AND ("conduit"[All Fields] OR "conduit s"[All Fields] OR "conduits"[All Fields])) OR (("outflow"[All Fields] OR "outflowing"[All Fields] OR "outflows"[All Fields]) AND ("tract"[All Fields] OR "tract s"[All Fields] OR "tracts"[All Fields]) AND ("plastic surgery procedures"[MeSH Terms] OR ("plastic"[All Fields] AND "surgery"[All Fields] AND "procedures"[All Fields]) OR "plastic surgery procedures"[All Fields] OR "reconstruction"[All Fields] OR "reconstructions"[All Fields] OR "reconstruct"[All Fields] OR "reconstructability"[All Fields] OR "reconstructable"[All Fields] OR "reconstructed"[All Fields] OR "reconstructible"[All Fields] OR "reconstructing"[All Fields] OR "reconstructional"[All Fields] OR "reconstructive"[All Fields] OR "reconstructs"[All Fields]))) AND (("non"[All Fields] AND ("conduit"[All Fields] OR "conduit s"[All Fields] OR "conduits"[All Fields])) OR "non-conduit"[All Fields] OR (("conduit"[All Fields] OR "conduit s"[All Fields] OR "conduits"[All Fields]) AND "free"[All Fields]) OR "conduit-free"[All Fields] OR (("direct"[All Fields] OR "directed"[All Fields] OR "directing"[All Fields] OR "direction compound"[Supplementary Concept] OR "direction compound"[All Fields] OR "direction"[All Fields] OR "directional"[All Fields] OR "directions"[All Fields] OR "directivities"[All Fields] OR "directivity"[All Fields] OR "directs"[All Fields]) AND "RV"[All Fields] AND ("pathology"[MeSH Subheading] OR "pathology"[All Fields] OR "pa"[All Fields])) OR (("direct"[All Fields] OR "directed"[All Fields] OR "directing"[All Fields] OR "direction compound"[Supplementary Concept] OR "direction compound"[All Fields] OR "direction"[All Fields] OR "directional"[All Fields] OR "directions"[All Fields] OR "directivities"[All Fields] OR "directivity"[All Fields] OR "directs"[All Fields]) AND "RVPA"[All Fields]) OR ("RVPA"[All Fields] AND ("conduit"[All Fields] OR "conduit s"[All Fields] OR "conduits"[All Fields])) OR (("indigenous peoples"[MeSH Terms] OR ("indigenous"[All Fields] AND "peoples"[All Fields]) OR "indigenous peoples"[All Fields] OR "natives"[All Fields] OR "native"[All Fields] OR "native s"[All Fields] OR "natively"[All Fields] OR "nativeness"[All Fields] OR "nativity"[All Fields]) AND ("connect"[All Fields] OR "connectable"[All Fields] OR "connected"[All Fields] OR "connecting"[All Fields] OR "connection"[All Fields] OR "connectional"[All Fields] OR "connections"[All Fields] OR "connective"[All Fields] OR "connectives"[All Fields] OR "connectivities"[All Fields] OR "connectivity"[All Fields] OR "connects"[All Fields] OR "connexion"[All Fields] OR "connexions"[All Fields])) OR (("indigenous peoples"[MeSH Terms] OR ("indigenous"[All Fields] AND "peoples"[All Fields]) OR "indigenous peoples"[All Fields] OR "natives"[All Fields] OR "native"[All Fields] OR "native s"[All Fields] OR "natively"[All Fields] OR "nativeness"[All Fields] OR "nativity"[All Fields]) AND ("anastomosis, surgical"[MeSH Terms] OR ("anastomosis"[All Fields] AND "surgical"[All Fields]) OR "surgical anastomosis"[All Fields] OR "anastomosis"[All Fields])) OR (("direct"[All Fields] OR "directed"[All Fields] OR "directing"[All Fields] OR "direction compound"[Supplementary Concept] OR "direction compound"[All Fields] OR "direction"[All Fields] OR "directional"[All Fields] OR "directions"[All Fields] OR "directivities"[All Fields] OR "directivity"[All Fields] OR "directs"[All Fields]) AND ("anastomosis, surgical"[MeSH Terms] OR ("anastomosis"[All Fields] AND "surgical"[All Fields]) OR "surgical anastomosis"[All Fields] OR "anastomosis"[All Fields])) OR (("heart ventricles"[MeSH Terms] OR ("heart"[All Fields] AND "ventricles"[All Fields]) OR "heart ventricles"[All Fields] OR ("right"[All Fields] AND "ventricle"[All Fields]) OR "right ventricle"[All Fields]) AND ("pulmonary artery"[MeSH Terms] OR ("pulmonary"[All Fields] AND "artery"[All Fields]) OR "pulmonary artery"[All Fields]) AND ("connect"[All Fields] OR "connectable"[All Fields] OR "connected"[All Fields] OR "connecting"[All Fields] OR "connection"[All Fields] OR "connectional"[All Fields] OR "connections"[All Fields] OR "connective"[All Fields] OR "connectives"[All Fields] OR "connectivities"[All Fields] OR "connectivity"[All Fields] OR "connects"[All Fields] OR "connexion"[All Fields] OR "connexions"[All Fields])))  **Translations**  **truncus arteriosus:** "truncus arteriosus"[MeSH Terms] OR ("truncus"[All Fields] AND "arteriosus"[All Fields]) OR "truncus arteriosus"[All Fields]  **common arterial trunk:** "truncus arteriosus, persistent"[MeSH Terms] OR ("truncus"[All Fields] AND "arteriosus"[All Fields] AND "persistent"[All Fields]) OR "persistent truncus arteriosus"[All Fields] OR ("common"[All Fields] AND "arterial"[All Fields] AND "trunk"[All Fields]) OR "common arterial trunk"[All Fields]  **persistent:** "persist"[All Fields] OR "persistance"[All Fields] OR "persistant"[All Fields] OR "persisted"[All Fields] OR "persistence"[All Fields] OR "persistences"[All Fields] OR "persistencies"[All Fields] OR "persistency"[All Fields] OR "persistent"[All Fields] OR "persistently"[All Fields] OR "persistents"[All Fields] OR "persister"[All Fields] OR "persisters"[All Fields] OR "persisting"[All Fields] OR "persists"[All Fields]  **truncus:** "torso"[MeSH Terms] OR "torso"[All Fields] OR "truncus"[All Fields]  **truncus:** "torso"[MeSH Terms] OR "torso"[All Fields] OR "truncus"[All Fields]  **congenital heart disease:** "heart defects, congenital"[MeSH Terms] OR ("heart"[All Fields] AND "defects"[All Fields] AND "congenital"[All Fields]) OR "congenital heart defects"[All Fields] OR ("congenital"[All Fields] AND "heart"[All Fields] AND "disease"[All Fields]) OR "congenital heart disease"[All Fields]  **congenital heart defect:** "heart defects, congenital"[MeSH Terms] OR ("heart"[All Fields] AND "defects"[All Fields] AND "congenital"[All Fields]) OR "congenital heart defects"[All Fields] OR ("congenital"[All Fields] AND "heart"[All Fields] AND "defect"[All Fields]) OR "congenital heart defect"[All Fields]  **anomaly:** "abnormalities"[Subheading] OR "abnormalities"[All Fields] OR "anomalies"[All Fields] OR "anomalie"[All Fields] OR "anomaly"[All Fields]  **single:** "single person"[MeSH Terms] OR ("single"[All Fields] AND "person"[All Fields]) OR "single person"[All Fields] OR "single"[All Fields] OR "singles"[All Fields]  **arterial:** "arterialization"[All Fields] OR "arterializations"[All Fields] OR "arterialize"[All Fields] OR "arterialized"[All Fields] OR "arterializing"[All Fields] OR "arterially"[All Fields] OR "arterials"[All Fields] OR "arterie"[All Fields] OR "arteries"[MeSH Terms] OR "arteries"[All Fields] OR "arterial"[All Fields] OR "arteris"[All Fields] OR "artery"[All Fields] OR "arterious"[All Fields] OR "artery's"[All Fields] OR "arterys"[All Fields]  **trunk:** "torso"[MeSH Terms] OR "torso"[All Fields] OR "trunk"[All Fields] OR "trunk's"[All Fields] OR "trunks"[All Fields]  **cyanotic:** "cyanotic"[All Fields] OR "cyanotics"[All Fields]  **congenital heart disease:** "heart defects, congenital"[MeSH Terms] OR ("heart"[All Fields] AND "defects"[All Fields] AND "congenital"[All Fields]) OR "congenital heart defects"[All Fields] OR ("congenital"[All Fields] AND "heart"[All Fields] AND "disease"[All Fields]) OR "congenital heart disease"[All Fields]  **conduit:** "conduit"[All Fields] OR "conduit's"[All Fields] OR "conduits"[All Fields]  **homograft:** "allografts"[MeSH Terms] OR "allografts"[All Fields] OR "homograft"[All Fields] OR "homografts"[All Fields]  **graft:** "graft's"[All Fields] OR "grafted"[All Fields] OR "graftings"[All Fields] OR "transplantation"[Subheading] OR "transplantation"[All Fields] OR "grafting"[All Fields] OR "transplantation"[MeSH Terms] OR "grafts"[All Fields] OR "transplants"[MeSH Terms] OR "transplants"[All Fields] OR "graft"[All Fields]  **valved:** "valve"[All Fields] OR "valve's"[All Fields] OR "valved"[All Fields] OR "valves"[All Fields] OR "valving"[All Fields]  **conduit:** "conduit"[All Fields] OR "conduit's"[All Fields] OR "conduits"[All Fields]  **graft:** "graft's"[All Fields] OR "grafted"[All Fields] OR "graftings"[All Fields] OR "transplantation"[Subheading] OR "transplantation"[All Fields] OR "grafting"[All Fields] OR "transplantation"[MeSH Terms] OR "grafts"[All Fields] OR "transplants"[MeSH Terms] OR "transplants"[All Fields] OR "graft"[All Fields]  **vascular graft:** "vascular grafting"[MeSH Terms] OR ("vascular"[All Fields] AND "grafting"[All Fields]) OR "vascular grafting"[All Fields] OR ("vascular"[All Fields] AND "graft"[All Fields]) OR "vascular graft"[All Fields]  **synthetic:** "synthetic"[All Fields] OR "synthetically"[All Fields] OR "synthetics"[All Fields] OR "synthetize"[All Fields] OR "synthetized"[All Fields] OR "synthetizing"[All Fields]  **conduit:** "conduit"[All Fields] OR "conduit's"[All Fields] OR "conduits"[All Fields]  **prosthetic:** "prosthetic"[All Fields] OR "prosthetically"[All Fields] OR "prosthetics"[All Fields]  **graft:** "graft's"[All Fields] OR "grafted"[All Fields] OR "graftings"[All Fields] OR "transplantation"[Subheading] OR "transplantation"[All Fields] OR "grafting"[All Fields] OR "transplantation"[MeSH Terms] OR "grafts"[All Fields] OR "transplants"[MeSH Terms] OR "transplants"[All Fields] OR "graft"[All Fields]  **PA:** "pathology"[Subheading] OR "pathology"[All Fields] OR "pa"[All Fields]  **conduit:** "conduit"[All Fields] OR "conduit's"[All Fields] OR "conduits"[All Fields]  **conduit:** "conduit"[All Fields] OR "conduit's"[All Fields] OR "conduits"[All Fields]  **outflow:** "outflow"[All Fields] OR "outflowing"[All Fields] OR "outflows"[All Fields]  **tract:** "tract"[All Fields] OR "tract's"[All Fields] OR "tracts"[All Fields]  **reconstruction:** "plastic surgery procedures"[MeSH Terms] OR ("plastic"[All Fields] AND "surgery"[All Fields] AND "procedures"[All Fields]) OR "plastic surgery procedures"[All Fields] OR "reconstruction"[All Fields] OR "reconstructions"[All Fields] OR "reconstruct"[All Fields] OR "reconstructability"[All Fields] OR "reconstructable"[All Fields] OR "reconstructed"[All Fields] OR "reconstructible"[All Fields] OR "reconstructing"[All Fields] OR "reconstructional"[All Fields] OR "reconstructive"[All Fields] OR "reconstructs"[All Fields]  **conduit:** "conduit"[All Fields] OR "conduit's"[All Fields] OR "conduits"[All Fields]  **conduit:** "conduit"[All Fields] OR "conduit's"[All Fields] OR "conduits"[All Fields]  **direct:** "direct"[All Fields] OR "directed"[All Fields] OR "directing"[All Fields] OR "direction compound"[Supplementary Concept] OR "direction compound"[All Fields] OR "direction"[All Fields] OR "directional"[All Fields] OR "directions"[All Fields] OR "directivities"[All Fields] OR "directivity"[All Fields] OR "directs"[All Fields]  **PA:** "pathology"[Subheading] OR "pathology"[All Fields] OR "pa"[All Fields]  **direct:** "direct"[All Fields] OR "directed"[All Fields] OR "directing"[All Fields] OR "direction compound"[Supplementary Concept] OR "direction compound"[All Fields] OR "direction"[All Fields] OR "directional"[All Fields] OR "directions"[All Fields] OR "directivities"[All Fields] OR "directivity"[All Fields] OR "directs"[All Fields]  **conduit:** "conduit"[All Fields] OR "conduit's"[All Fields] OR "conduits"[All Fields]  **native:** "indigenous peoples"[MeSH Terms] OR ("indigenous"[All Fields] AND "peoples"[All Fields]) OR "indigenous peoples"[All Fields] OR "natives"[All Fields] OR "native"[All Fields] OR "native's"[All Fields] OR "natively"[All Fields] OR "nativeness"[All Fields] OR "nativity"[All Fields]  **connection:** "connect"[All Fields] OR "connectable"[All Fields] OR "connected"[All Fields] OR "connecting"[All Fields] OR "connection"[All Fields] OR "connectional"[All Fields] OR "connections"[All Fields] OR "connective"[All Fields] OR "connectives"[All Fields] OR "connectivities"[All Fields] OR "connectivity"[All Fields] OR "connects"[All Fields] OR "connexion"[All Fields] OR "connexions"[All Fields]  **native:** "indigenous peoples"[MeSH Terms] OR ("indigenous"[All Fields] AND "peoples"[All Fields]) OR "indigenous peoples"[All Fields] OR "natives"[All Fields] OR "native"[All Fields] OR "native's"[All Fields] OR "natively"[All Fields] OR "nativeness"[All Fields] OR "nativity"[All Fields]  **anastomosis:** "anastomosis, surgical"[MeSH Terms] OR ("anastomosis"[All Fields] AND "surgical"[All Fields]) OR "surgical anastomosis"[All Fields] OR "anastomosis"[All Fields]  **direct:** "direct"[All Fields] OR "directed"[All Fields] OR "directing"[All Fields] OR "direction compound"[Supplementary Concept] OR "direction compound"[All Fields] OR "direction"[All Fields] OR "directional"[All Fields] OR "directions"[All Fields] OR "directivities"[All Fields] OR "directivity"[All Fields] OR "directs"[All Fields]  **anastomosis:** "anastomosis, surgical"[MeSH Terms] OR ("anastomosis"[All Fields] AND "surgical"[All Fields]) OR "surgical anastomosis"[All Fields] OR "anastomosis"[All Fields]  **right ventricle:** "heart ventricles"[MeSH Terms] OR ("heart"[All Fields] AND "ventricles"[All Fields]) OR "heart ventricles"[All Fields] OR ("right"[All Fields] AND "ventricle"[All Fields]) OR "right ventricle"[All Fields]  **pulmonary artery:** "pulmonary artery"[MeSH Terms] OR ("pulmonary"[All Fields] AND "artery"[All Fields]) OR "pulmonary artery"[All Fields]  **connection:** "connect"[All Fields] OR "connectable"[All Fields] OR "connected"[All Fields] OR "connecting"[All Fields] OR "connection"[All Fields] OR "connectional"[All Fields] OR "connections"[All Fields] OR "connective"[All Fields] OR "connectives"[All Fields] OR "connectivities"[All Fields] OR "connectivity"[All Fields] OR "connects"[All Fields] OR "connexion"[All Fields] OR "connexions"[All Fields] |

**Supplementary Table 2.** Demographic data of the patient population

| **Author** | **Patients,**  **n (%)** | | **Age,**  **days** | | **Male,**  **n (%)** | | **Weight,**  **kg** | | **Truncal Stenosis/Insufficiency**  **n (%)** | | **Type I/II**  **TA**  **n (%)** | | **Type III/IV**  **TA**  **n (%)** | | **IAA**  **n (%)** | | | | **CA**  **Anomalies**  **n (%)** | | | |
| --- | --- | --- | --- | --- | --- | --- | --- | --- | --- | --- | --- | --- | --- | --- | --- | --- | --- | --- | --- | --- | --- | --- |
|  | **C** | **DA** | **C** | **DA** | **C** | **DA** | **C** | **DA** | **C** | **DA** | **C** | **DA** | **C** | **DA** | **C** | | | **DA** | **C** | | | **DA** |
| Brown | 54 (90) | 6 (10) | 76  (3-20)* | | 22  (37) | | - | - | 7  (12) | | 52  (86) | | 4  (7) | | 6  (10) | | | | 6  (10) | | | |
| Chen | 15 (28) | 39 (72) | 16  (4-159) * | | - | | - | - | - | - | 50  (93) | | 4  (7) | | - | - | | | 8  (15) | | | |
| Danton | 38 (62) | 23 (38) | 34  (2-192)* | | 30  (77) | | 3.2  (1.7-8.6)* | | 17  (28) | | - | | - | - | 7  (11) | | | | 9  (15) | | | |
| Derridji | 75 (60) | 50 (40) | 39.6  (32) ^§^ | | 34  (45) | 27  (54) | 3.36  (0.51) ^§^ | | 26  (35) | 27  (54) | 59  (79) | 44  (88) | 4  (5.3) | 1  (1.9) | 12  (16) | 8  (16) | | | 7  (9.3) | 11  (22) | | |
| Lacour-Gayet | 56  (50) | | 41  (2-240)* | | - | - | 3.4  (2.8-6.5)* | | 2  (4) | | 42  (75) | | 13  (23) | 1  (2) | 9  (16) | | | | - | | | |
| Luo | 54 (51) | 51 (49) | 14.8  (9.2) ^§^ | | 61  (58) | | 6.9  (2.3) ^§^ | | 54  (51.4) | | - | | - | - | 13  (12.4) | | | | 18  (17.1) | | | |
| Moodley | 34 (63) | 20 (37) | 91 (58-163.5) * | 40.5 (28-59) * | 18 (47) | 9  (45) | 3.9 (3.3-5.0)* | 3.1 (2.7-3.4)* | 8  (15) | | - | | -  - | - | - | | - | | - | | - | |
| Padalino | 17 (59) | 12 (41) | 24 (7-2104)* | 32  (7-303)* | 9  (53) | 5  (42) | - | - | 6  (35) | 5  (42) | - | | -  - | - | - | | - | | 8  (47) | | 2  (17) | |
| Raisky | 15 (47) | 17 (53) | 90 (69)^§^ | 54 (65)^§^ | 16  (50) | | 3.8 (1.4)^§^ | 3.3 (0.7)^§^ | - | - | -  - | | 3  (20) | 4  (24) | 3 (20) | | 4 (24) | | 4  (27) | | 3  (18) | |
| Reddy | 56  (50) | | 34.5  (18–73)^†^ | | 27  (48.2) | | 9.35  (7–14) ^†^ | | 12  (21.5) | | 27  (48) | 26  (46) | 3  (5.4) | | 2  (3.5) | | | | 3  (5.3) | | | |
| Xu | 5  (22) | 18 (78) | 608 (703) ^§^ | 213  (219) ^§^ | - | - | 6.3  (2.7) ^§^ | | 10  (43.5) | | - | | - | - | 3  (13) | | | | 4  (17.4) | | | |

**VC = Valved Conduit, DA = Direct Anastomosis, I/C = Intervention/Comparator, TA = Truncus Arteriosus, IAA = Interrupted Aortic Arch, CA anomalies = Coronary Artery anomalies, kg = kilogram, n = number, with data presented as mean (standard deviation)§, median (range)*, or median (interquartile range)^†^.**

**Supplementary Table 3.** Demographic data of the patient population (Weighted Averages)

| **Characteristics** | **Patients,**  **n** | **Weighted Average** |
| --- | --- | --- |
| **Age,**  **days** | 285 | 54.9 |
| **Weight,**  **kg** | 285 | 4.9 |

**Kg = kilogram, n = number, with data presented as weighted means.**

**Supplementary Table 4.** ROBINS-1 Risk of Bias assessment

**
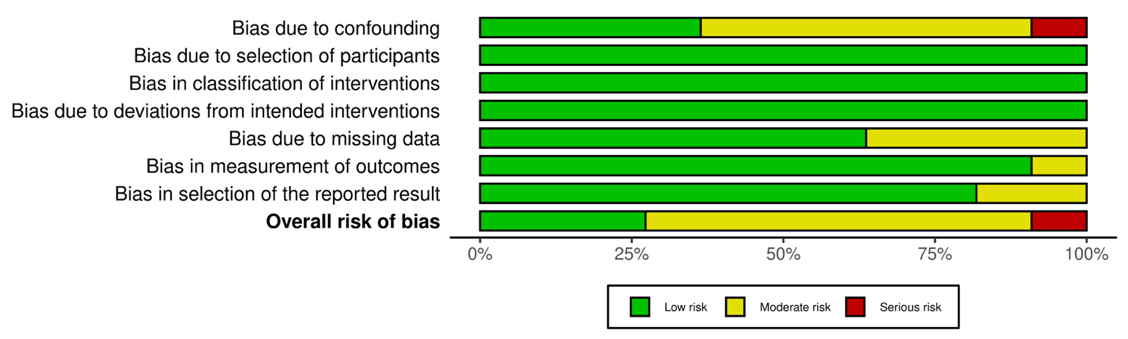

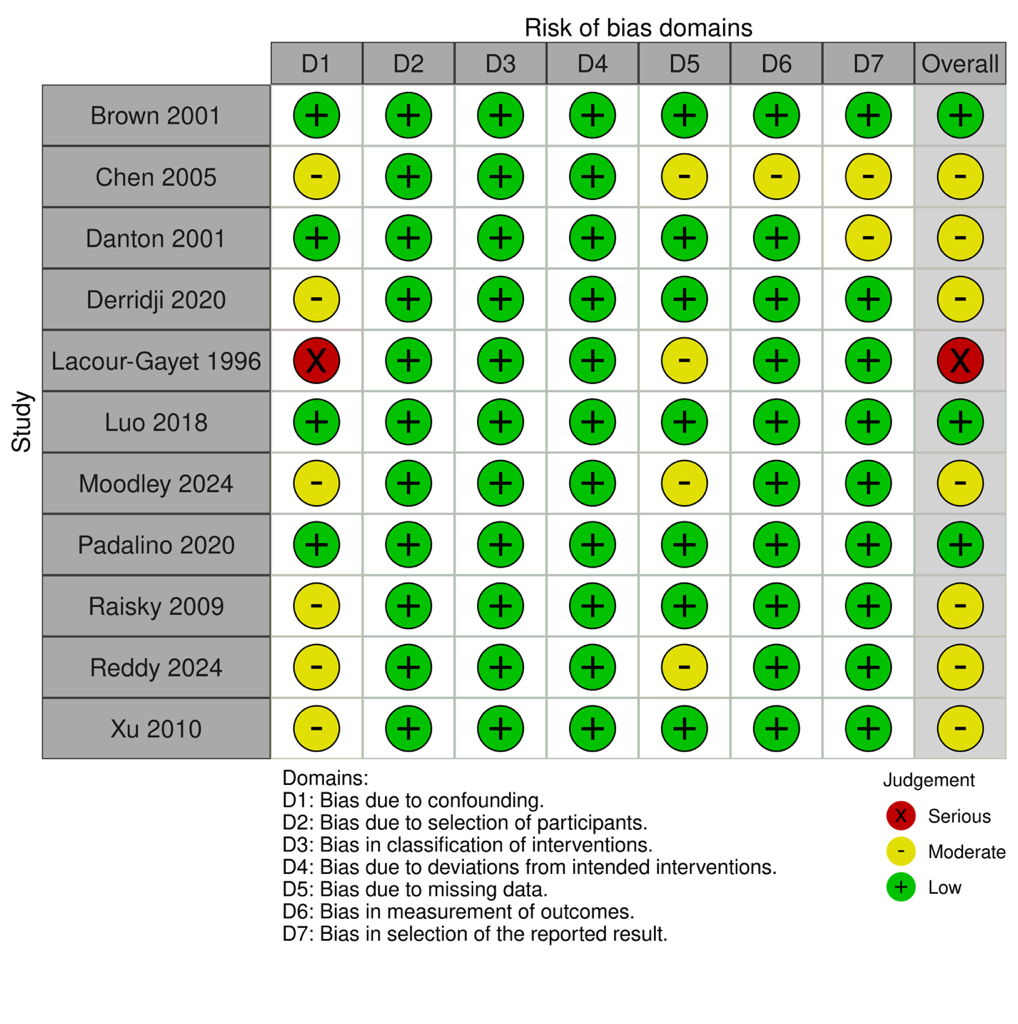
**

**Supplementary Table 5.** Meta regression of 30-day mortality.

| **Predictor** | **Coefficient** | **Standard Error** | **p-value** |
| --- | --- | --- | --- |
| **Year** | 0.14 | 0.29 | 0.35 |
| **Age (days)** | 0.03 | 0.16 | 0.73 |

**Supplementary Table 6.** Meta regression of Incidence of Surgical Reoperation.

| **Predictor** | **Coefficient** | **Standard Error** | **p-value** |
| --- | --- | --- | --- |
| **Year** | 0.48 | 0.51 | 0.19 |
| **Age (days)** | 0.03 | 0.22 | 0.80 |

**Supplementary Figure 1.** Publication Bias Assessment – Funnel Plot of the early mortality.

**
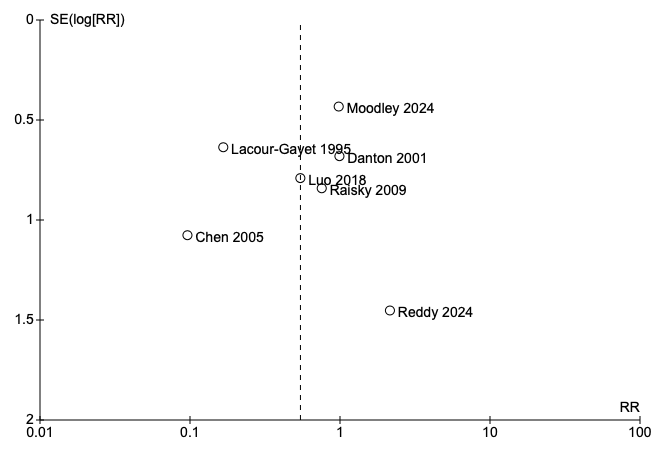
**

**Supplementary Figure 2.** Forest plot for Postoperative Surgical Reoperation.

**
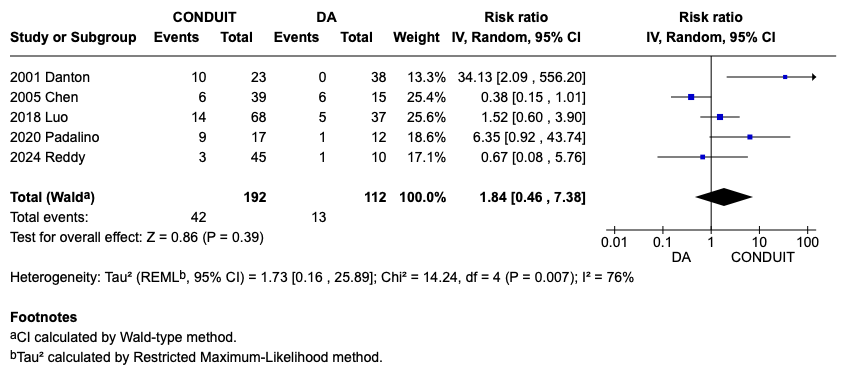
**

**Supplementary Figure 3.** Cardiopulmonary bypass time.


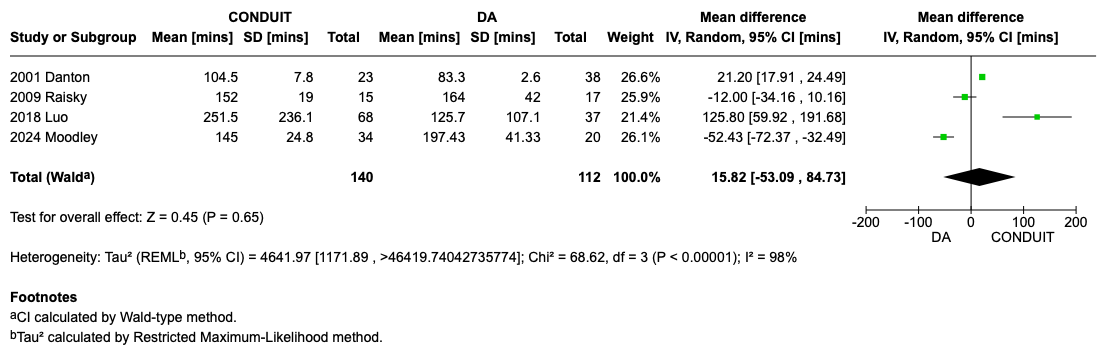


**Supplementary Figure 4.** Aortic cross-clamp time.


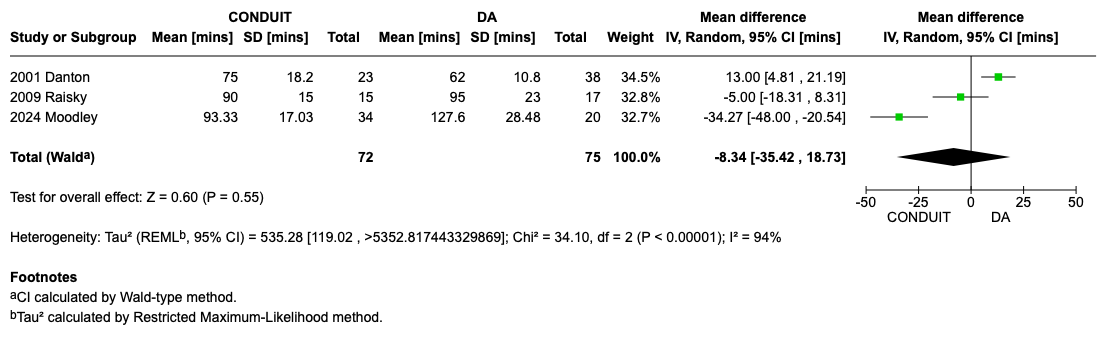


**Supplementary Figure 5.** ICU length of stay.


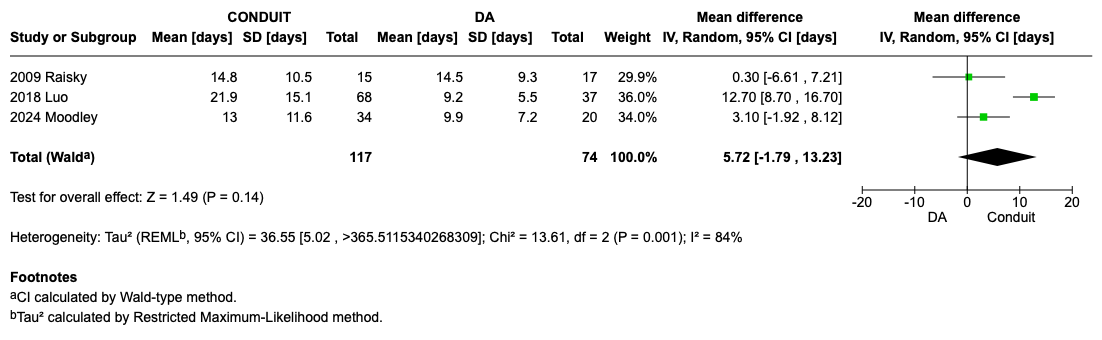


**Supplementary Figure 6.** Hospital length of stay.


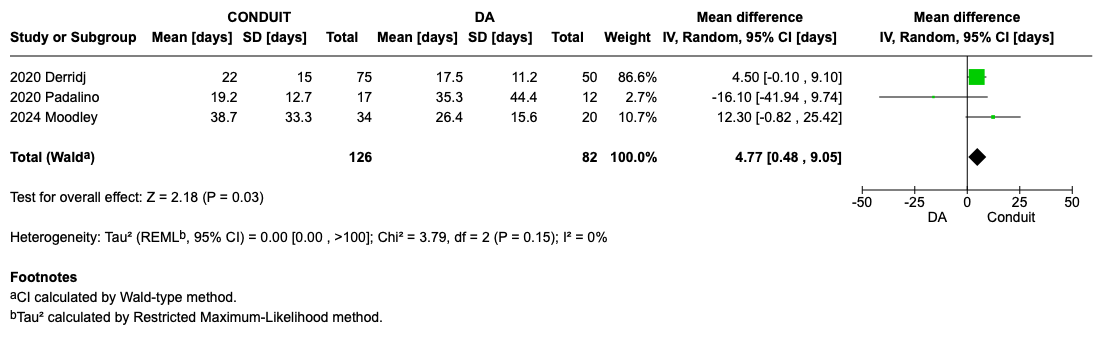


**Supplementary Figure 7.** Mechanical Ventilation Duration.


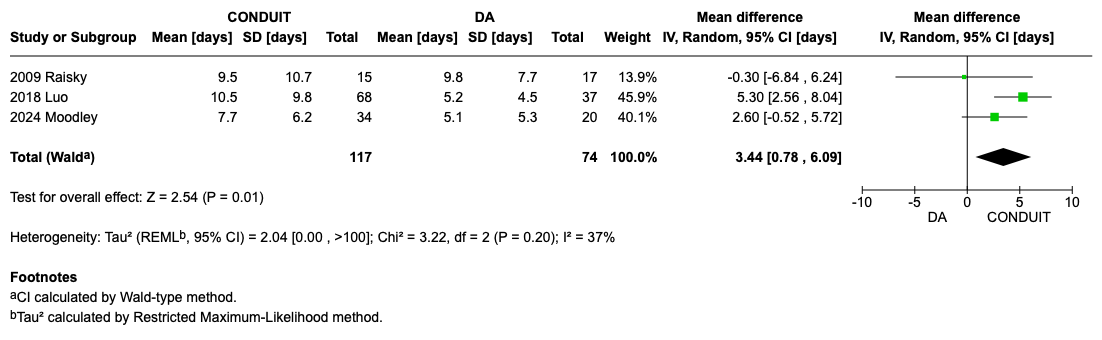


**Supplementary Figure 8.** RVOT growth.


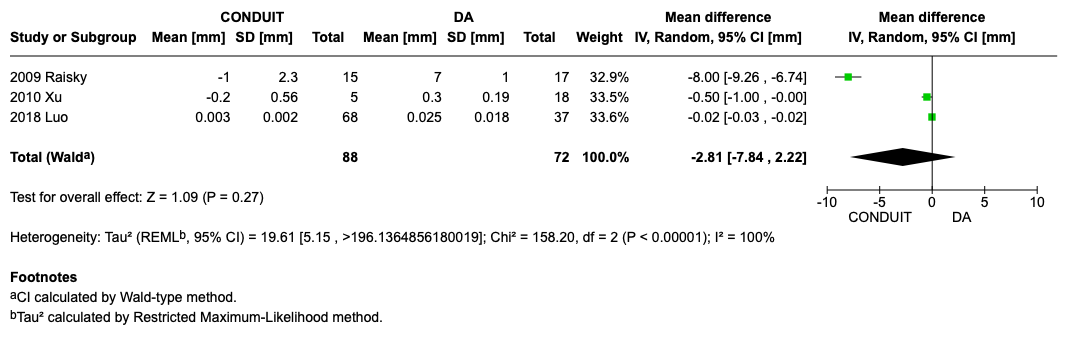


**Supplementary Figure 9.** Postoperative Truncal Valve Regurgitation.


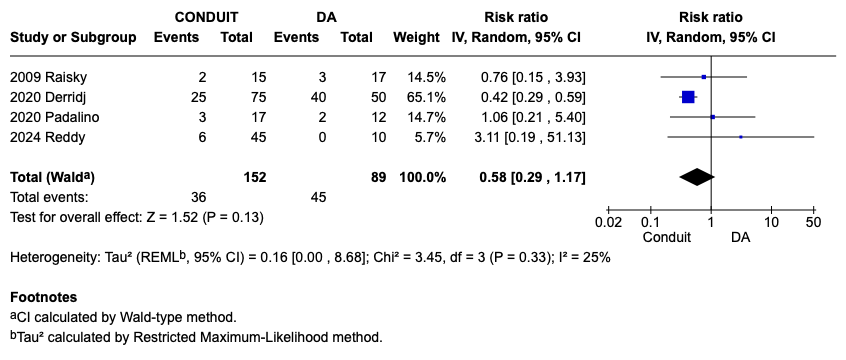


**Supplementary Figure 10.** Meta-regression plot for Mortality and Year.

**
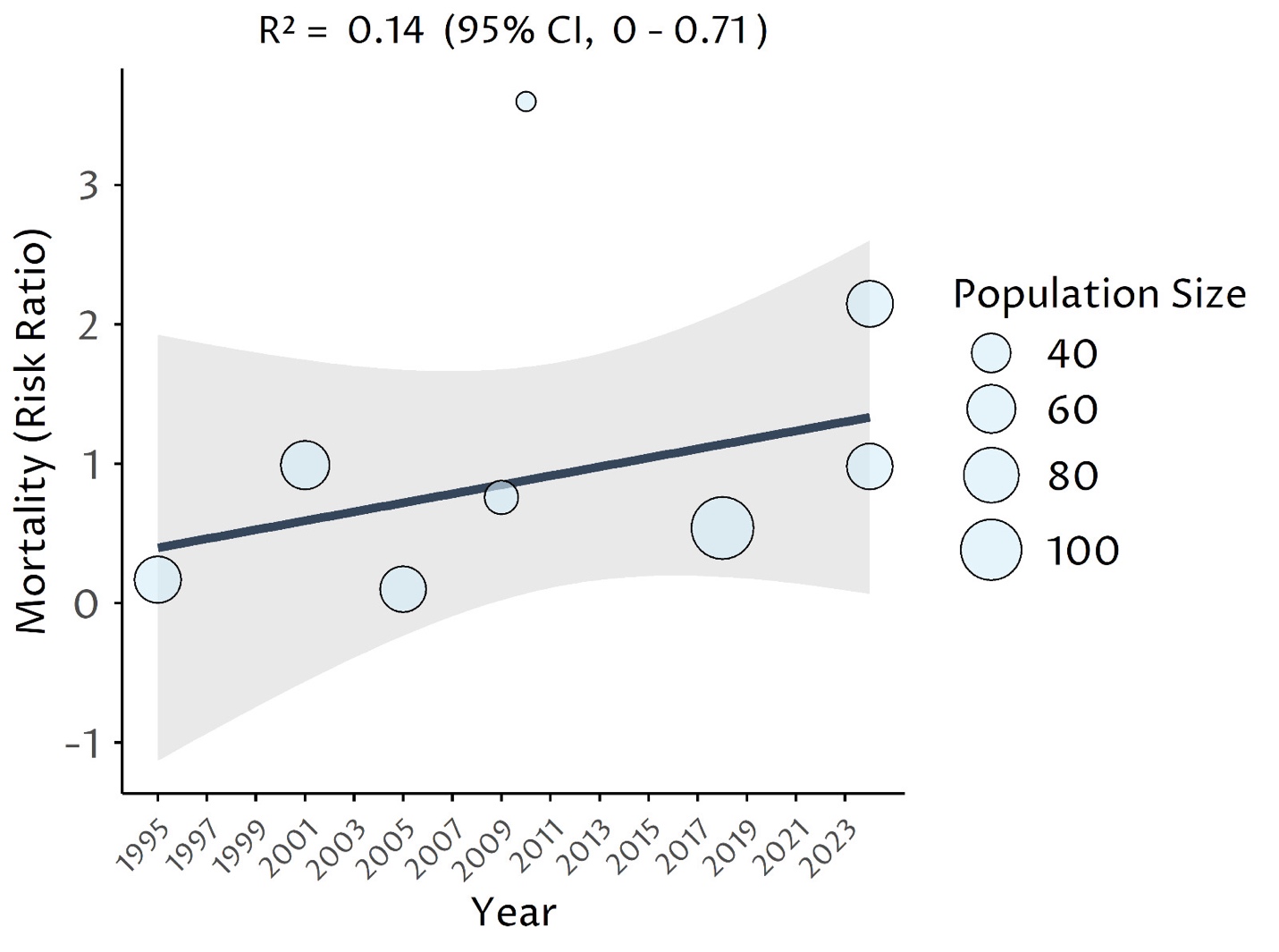
**

**Supplementary Figure 11.** Meta-regression plot for Mortality and Age.

**
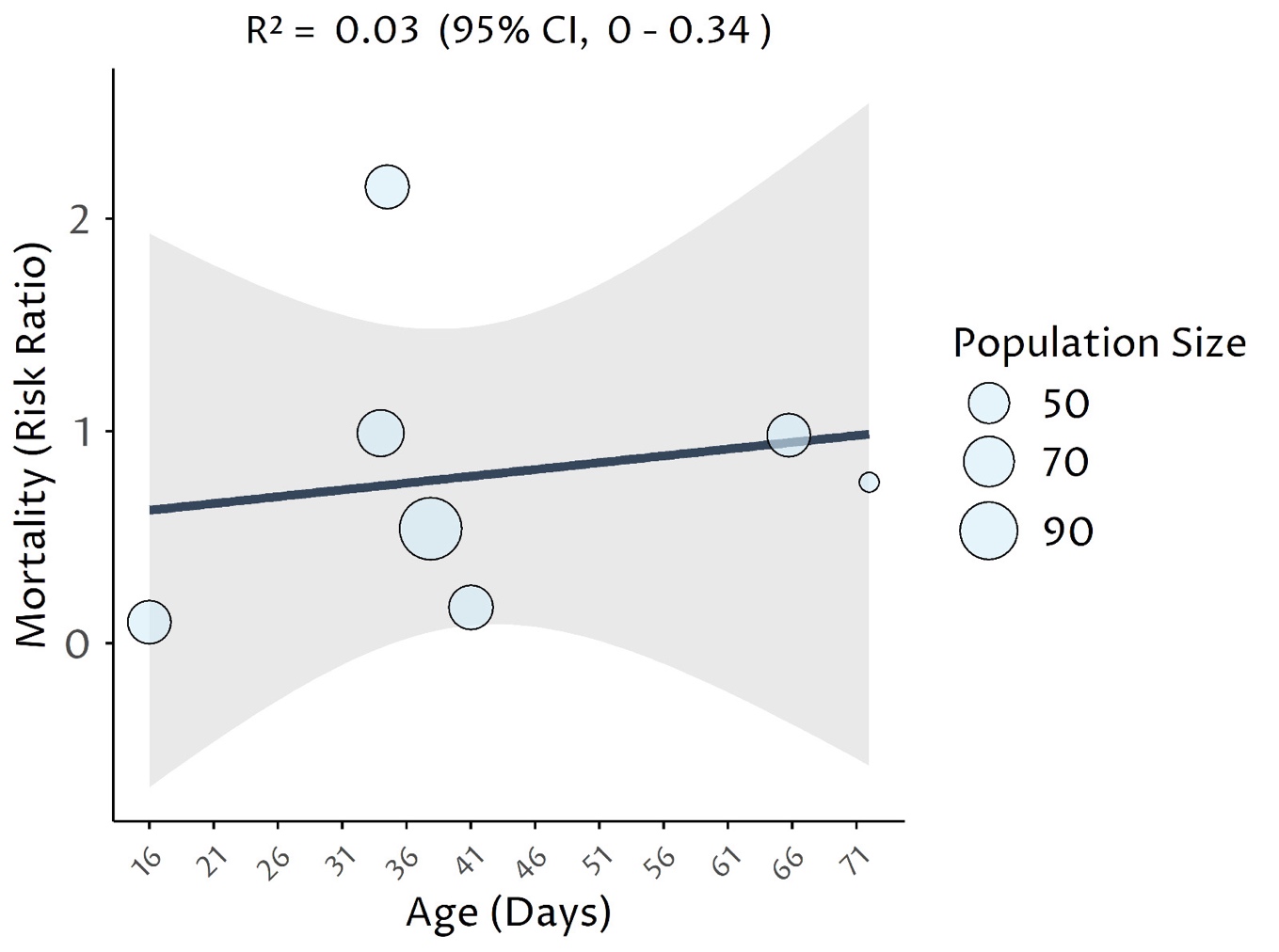
**

**Supplementary Figure 12.** Meta-regression plot for Incidence of Surgical Reoperation and Year.

**
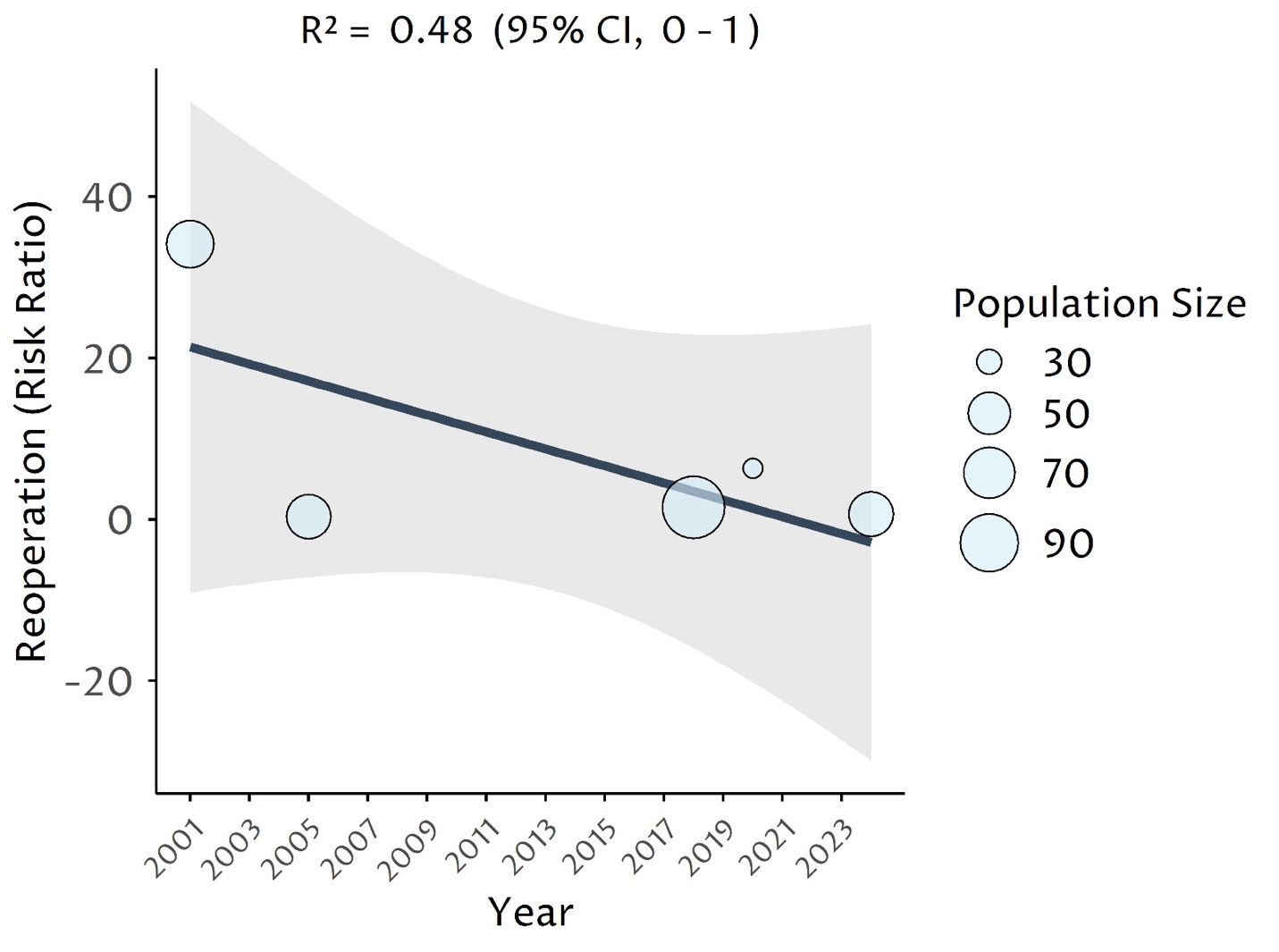
**

**Supplementary Figure 13.** Meta-regression plot for Incidence of Surgical Reoperation and Age.


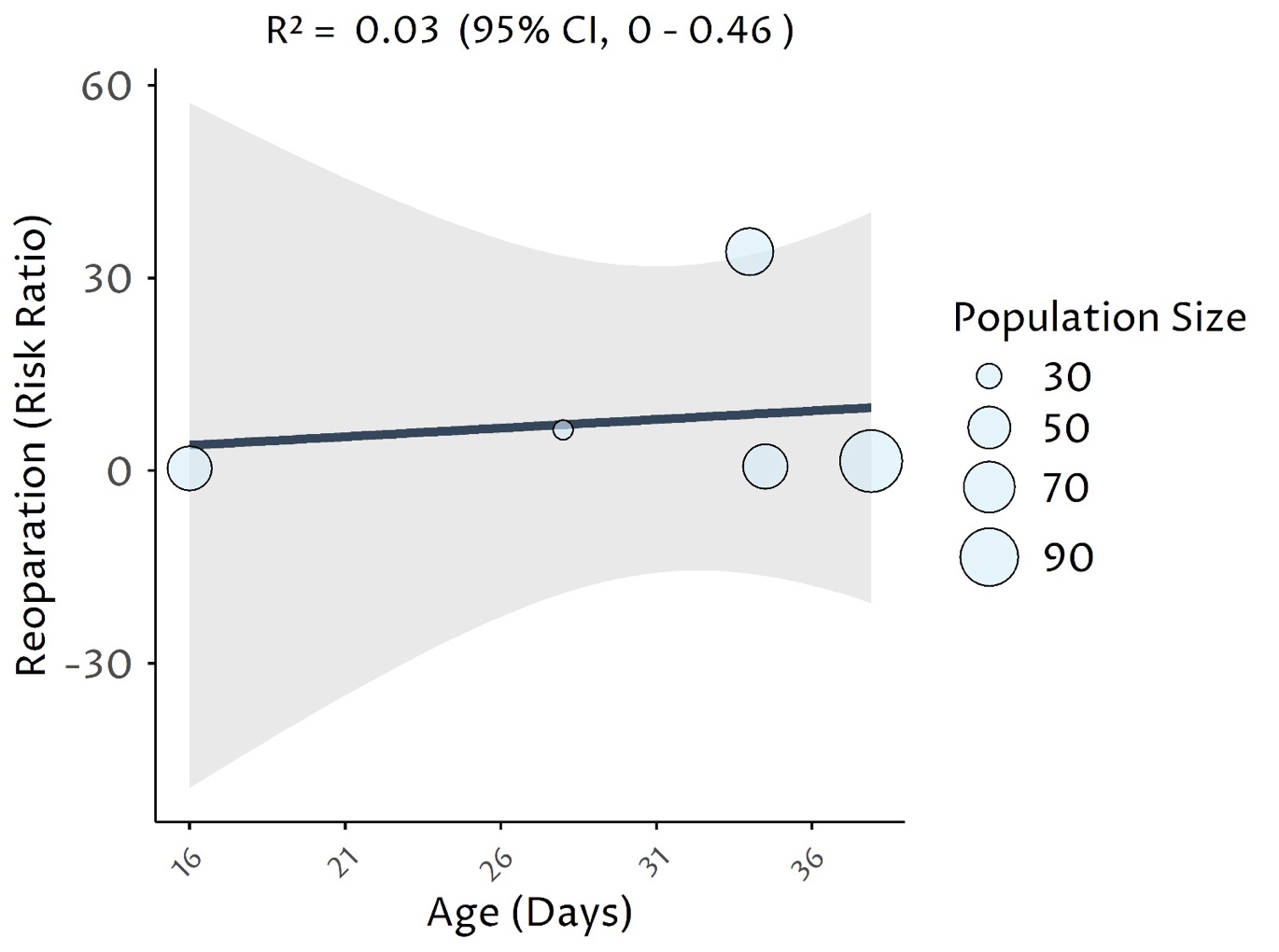

Supplement: ivag029_Supplementary_Data [file ivag029_supplementary_data.zip › Supplementary Material File - Conduit vs DAT. Revised.docx]
